# Inorganic–Organic Multicoating Layer Encapsulation of Formamidine Lead Halide Perovskite Quantum Dots for Lighting Applications

**DOI:** 10.1021/acsami.5c24129

**Published:** 2026-02-11

**Authors:** Ling Hsuan Chung, Andi Magattang Gafur Muchlis, Po-Chun Li, Yan Chung Lai, Yuan-Hong Chen, Jung-An Cheng, Chun Che Lin

**Affiliations:** † Institute of Organic and Polymeric Materials, National Taipei University of Technology, Taipei 106334, Taiwan; ‡ Research and Development Center for Smart Textile Technology, 34877 National Taipei University of Technology, Taipei 106334, Taiwan; § Foxconn Technology Co., Ltd,, Miaoli County 35053, Taiwan

**Keywords:** formamidinium lead bromide, perovskite quantum dots, multicoating layer, inorganic SiO_*x*_, dicyclopentanyl methacrylate

## Abstract

Pure-green formamidinium lead bromide (FAPbBr_3_) perovskite quantum dots (PQDs) are particularly attractive for display and lighting applications. However, their inherent instability and processing challenges hinder their widespread application and commercialization. The instability of PQDs under exposure to light, heat, water, and oxygen is primarily attributed to their low formation energy, leading to phase transformations, agglomeration, and degradation, which negatively impact their optical properties. To address these challenges, this study proposes a dual-interface encapsulation strategy that integrates inorganic–organic synergy and covalent surface coupling into a single hierarchical framework. In this work, we present a cost-effective hierarchical multicoating strategy for stabilizing pure-green FAPbBr_3_ PQDs using industrially accessible stabilization agents, namely SiO_*x*_ and dicyclopentanyl methacrylate (513M). Specifically, this research utilizes (3-aminopropyl) triethoxysilane (APTES) as a coupling agent ligand and tetraethoxysilane to uniformly coat the PQDs by SiO_*x*_. Following this, 513M, a monomer, is radically polymerized on the surface of the SiO_*x*_-coated PQDs to form a secondary shell layer. The initial coating enhances the PQDs’ resistance to environmental factors, while the secondary layer (a hydrophobic polymer) further improves environmental stability without compromising the PQDs' structure during polymerization. The resulting FAPbBr_3_@SiO_*x*_@513M composite material, resulted in powder form, significantly improves the PQDs’ durability against environmental conditions while maintaining excellent optical properties, including emission at ∼532 nm, a full width at half-maximum of ≤28 nm, and a photoluminescence quantum yield of >50%, demonstrating that robust environmental protection can be achieved without relying on record-high optical parameters or costly materials. Owing to its use of low-cost, scalable materials and pure-green emissive PQDs, this multicoating strategy offers a realistic pathway toward industrially viable, solid-state PQD materials for optoelectronic applications.

## Graphical abstract

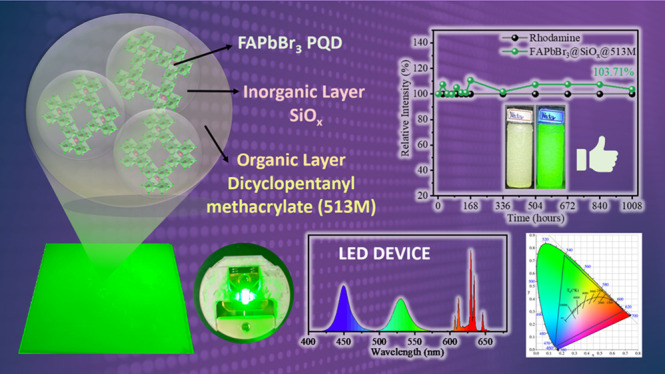

## Introduction

In recent years, the optoelectronics industry has approached saturation in its current development. To continue driving innovation and creating superior product experiences while meeting environmental, social, and governance (ESG) standards, such as green technology, low carbon emissions, energy efficiency, and low toxicity, the industry is exploring new technological advancements. These advancements include quantum technology, universal energy conversion techniques, and advanced optical materials to cater to the demands of cutting-edge applications.

Since the advent of perovskite quantum dots (PQDs) in 2015,^1^ their unique advantages, including high color purity, intense fluorescence, high photoluminescence quantum yield (PLQY), tunable energy gaps, high absorption coefficients, exceptional carrier mobility, and low synthesis difficulty, have received significant attention.^2–5^ However, PQDs face a major limitation: their low stability, which causes them to lose their original optical properties.^6–10^ Perovskite commonly structured as ABX_3_ formula, where A and B are cations and X is anions. The organic cations in PQD crystals, such as methylammonium (MA^+^), ethylammonium (EA^+^), and formamidinium (FA^+^), are particularly prone to instability due to their low formation energy, making them highly susceptible to environmental factors, resulting in reduction reactions or phase transitions. When exposed to high-energy electromagnetic waves, they generate electrons that react with O_2_ or CO_2_ in the environment, forming free radicals. These free radicals interact with the A-site cations, producing volatile gases that create structural vacancies and decompose the PQDs.^10–12^

Additionally, energy input can weaken the protective ligand shell on the PQDs surface, increasing surface defects. This leads to processes such as reconstruction between PQDs, ion migration,^13^ and Ostwald ripening,^14,15^ causing agglomeration into larger particles. Moreover, exposure to water and oxygen triggers affinity substitution reactions, reducing the B-site element in the octahedral core and releasing X-site halogen atoms as hydrogen halide gas. Thus, ultimately exacerbates the PQDs’ degradation.

Surface passivation of quantum dots is one of the most direct and effective protection strategies currently under research. By employing surface passivation techniques, surface defects can be minimized, enhancing the stability of quantum dots in diverse environmental conditions. Prolongs their lifespan while maintaining their exceptional optoelectronic properties. In previous research, many scientists attempted to stabilize PQDs with various passivation materials and encapsulation techniques. For instance, porous materials such as mesoporous silica nanoparticles (MSNs),^16–18^ metal–organic frameworks (MOFs),^19,20^ and even salicylic acid crystals^21^ have been employed to load and passivate PQDs, thereby enhancing their photoluminescence stability. Stable oxides (e.g., silicon oxide, aluminum oxide) have also been used recently to encapsulate PQDs, showing promising results for optical device applications.^22,23^ On the other hand, hydrophobic polymers such as poly­(methyl methacrylate) (PMMA) have been investigated to protect PQDs from water, oxygen, and humidity, thereby preserving their luminescence.^24^

Despite numerous research attempting to stabilize PQDs over the past few years, challenges related to cost, yield, and manufacturing complexity have hindered these methods’ application in commercial products. Therefore, this study reports an experimental method involving dual encapsulation of quantum dot materials, which maximizes the stability of PQDs while sacrificing only a minimal amount of quantum efficiency. Furthermore, the resulting material can be directly processed into many industry-ready optical devices.

Among green-emission PQDs (e.g., CsPbBr_3_, MAPbBr_3_, and FAPbBr_3_), FAPbBr_3_ exhibits superior optical quality, higher PLQY, and purer green color, with competitive stability when appropriately encapsulated, making it an excellent model system for our study.^25–27^ These advantages encourage us to explore more about this type of PQDs in this work.

Currently, several studies have reported double-layer SiO_2_ and polymer coatings for PQDs; however, these are largely limited to CsPbBr_3_, with comparatively little information available for FAPbBr_3_.^28–30^ In addition, the use of the hydrophobic organic monomer dicyclopentanyl methacrylate (513M) as the protecting polymer layer for PQDs remains underexplored. 513M was chosen because of its combination of hydrophobicity, rigidity, and chemical compatibility with the inorganic SiO_*x*_ shell. The bulky cyclopentanyl ring in 513M provides high mechanical stability and excellent resistance to moisture and oxygen, while the methacrylate group allows facile polymerization and strong interfacial adhesion with the SiO_*x*_ surface. In addition, 513M features a rigid bicyclic structure that results in a higher glass transition temperature, effectively preventing degradation of the encapsulating composite. After polymerization, it exhibits several advantageous properties, including low curing shrinkage, low volatility, and strong hydrophobicity. Furthermore, 513M can be processed under mild, solution-based conditions and forms an optically transparent coating, making it suitable for scalable encapsulation of PQDs.^31,32^ Therefore, in this work, we aim to investigate FAPbBr_3_ PQDs using a double-layer encapsulation strategy combining inorganic SiO_*x*_ and 513M polymer.

The synthesis process consists of three main steps: Synthesis of FAPbBr_3_ nanocrystals, which were synthesized by using a hot injection method. Then, SiO_*x*_ protective layer was generated on the quantum dot surface via hydrolysis–condensation. During this step, (3-aminopropyl) triethoxysilane (APTES) was introduced as a coupling agent. The NH^4+^ functional group on one end of APTES coordinates with the nanocrystal surface, while the SiOC_2_H_5_ group undergoes hydrolysis and condensation with other siloxanes to form SiO_*x*_, effectively anchoring the SiO_*x*_ layer onto the FAPbBr_3_ surface. After that, the organic hydrophobic monomer 513M was polymerized on the surface of FAPbBr_3_@SiO_*x*_ to form a second encapsulation layer. To facilitate practical handling, scalable processing, and integration into devices, the final FAPbBr_3_@SiO_*x*_@513M material was prepared in powder form using ball milling. This method offers high reproducibility, uniform particle size distribution down to the micrometer scale, and cost-effective scalability compared with alternative techniques, making it well-suited for industrial applications.^33^ The product exhibits exceptional stability under various challenging conditions, including polar solvents, light, heat, and the presence of additives. The material maintained 74.4% of its initial intensity after 336 h on a blue display panel and retained 65.1% of its initial intensity after heating at 60 °C for 336 h. Remarkably, when immersed in water, it completely preserved its initial intensity. Furthermore, during an 85 °C thermal cycling test, the material did not exhibit any permanent shifts in its emission wavelength. This advantage extends its potential for applications in extreme environments, providing users with the highest level of reliability.

While prior studies have primarily focused on surface passivation or single-shell encapsulation of PQDs (mostly CsPbBr_3_ systems), these protection strategies remain incomplete, either providing insufficient chemical shielding from inorganic shells or limited mechanical durability from polymer coatings.^6,8,16,23,30^ Moreover, such approaches are rarely designed from a dual-interface perspective that integrates inorganic–organic synergy with controlled surface chemical coupling.

In this study, we propose a generalizable dual-interface encapsulation framework that couples an inorganic SiO_*x*_ shell with a hydrophobic polymer layer of 513M through an APTES-mediated covalent interface. This design forms a continuous and chemically bonded protection barrier against both physical and chemical degradation pathways. Unlike previous incremental coating strategies, our approach introduces a hierarchical encapsulation architecture in which the inorganic SiO_*x*_ shell provides rigidity and ionic stability, while the polymeric 513M layer ensures hydrophobic sealing and mechanical flexibility.

Although FAPbBr_3_ was selected as the model system due to its superior green emission and intrinsic optical performance,^25–27^ the proposed encapsulation strategy is universally applicable to other halide perovskite families. The resulting FAPbBr_3_@SiO_*x*_@513M nanocomposite exhibits outstanding durability (retaining over 70% photoluminescence intensity after 336 h under various stress conditions) and highlights the potential of inorganic–organic hybrid protection as a scalable and industry-oriented solution for long-term stable PQD-based optoelectronic devices.

Importantly, this dual-encapsulation process employs low-cost precursors (TEOS, APTES, and 513M), mild reaction conditions, and a simple solution-based synthesis route that avoids complex vacuum or high-temperature processing, thereby ensuring both high yield and scalability. Furthermore, the resulting solid-state material can be directly integrated into a wide range of practical optical and optoelectronic applications.

## Experimental Section

### Materials

In this work, we purchased the listed chemicals below. Formamidine acetate (FAAc, Thermo scientific, 99%), lead­(II) bromide (PbBr_2_, Thermo scientific, 99.998%), oleic acid (OA, 90%, Sigma-Aldrich), oleyl amine (OAm, 70%, Sigma-Aldrich), 1-octadecene (ODE, 90%, Thermo scientific), (3-aminopropyl) triethoxysilane (APTES, 99%, Thermo scientific), tetraethoxysilane (TEOS, 98%, Acros), dicyclopentanyl methacrylate (513M, 96%, Resonac), diphenyl (2,4,6-trimethylbenzoyl) phosphine oxide (TPO, 97%, Merck), and hexane (≥98.5%, Macron).

### Synthesis of FAPbBr_3_ PQDs

In this process, a dual-precursor hot injection method was employed, where the dual precursors consisted of the A-site precursor (FA^+^ group) and the BX-site precursor (Pb^2+^ and Br^–^ ions). To prepare the FA precursor, 9.1 mmol of FAAc was dissolved in 15.8 mmol of OA. For the PbBr_2_ precursor, 0.27 mmol of PbBr_2_, 2.5 mmol of OA, and 1.5 mmol of OAm were added into 25 mmol of ODE. Both precursors were heated to 100 °C to dissolve the reagents in their respective solvents, with alternating vacuum and nitrogen purging to eliminate moisture and oxygen.

Subsequently, various mmol ratio of APTES was added to the PbBr_2_ precursor, and the degassing process was repeated to ensure complete removal of water and oxygen. Once dissolved, the FA precursor was heated to 120 °C, and the PbBr_2_ precursor to 150 °C. At this point, 1 mL of the FA precursor solution was injected into the PbBr_2_ precursor. The reaction proceeded for 8 s before the crystallization was quenched by immersing the mixture in an ice bath.

The resulting crude solution was centrifuged at 12000 rpm to remove the supernatant. Afterward, 5 mL of hexane was added, followed by centrifugation at 5000 rpm. The supernatant was collected to yield the purified FAPbBr_3_ PQDs solution.

### Synthesis of FAPbBr_3_@SiO_*x*_

5 mL of the purified FAPbBr_3_ PQDs solution was taken and transferred into a container. 80 mmol of hexane and 0.7 mmol of TEOS were then added to the solution and stirred at 300 rpm. The setup was placed in an environment with 50% room humidity (RH) and allowed to react for 24 h. During this period, hydrolysis and condensation reactions occurred, forming the FAPbBr_3_@SiO_*x*_ suspension.

### Synthesis of FAPbBr_3_@SiO_*x*_@513M

The FAPbBr_3_@SiO_*x*_ dispersion was mixed with 513M at a ratio of 1 mL dispersion to 0.6 g of 513M, with proportional scaling for subsequent batches. After that, we added 2% TPO as a photoinitiator, then the curing reaction was initiated using a 20W UV lamp at a wavelength of 365 nm. Once cured, the resulting FAPbBr_3_@SiO_*x*_@513M solid was ground into smaller granules using a mortar and pestle.

We transferred the ground powder into a zirconia milling jar containing 5 mm zirconia beads and ball milling at a frequency of 25 Hz for 20 min was performed. After milling, the material was sieved using an appropriately sized mesh to obtain uniform FAPbBr_3_@SiO_*x*_@513M powder.

### Reliability Test

The reliability test includes four types of assessments: light resistance, heat resistance, water resistance, and thermal cycling. In the light resistance test, all samples were placed in a sealed chamber simulating a mixed blue (445 nm) and red (630 nm) light display, with the light intensity set at 500 nits. The irradiance at the sample surface was 159 mW/cm^2^, and the photostability assessment was performed over a period of 0–504 h. Before each measurement, the samples were removed from the display panel and stored in a dark environment for 30 min to stabilize before data collection. In the heat resistance test, all samples were placed in a 60 °C oven. Before each measurement, they were kept in a dark and room-temperature environment for 30 min to stabilize before data collection. For the water resistance test, the sample powder was added to a cuvette, completely sealed, and stored in a dark and room-temperature environment, then measured. In the thermal cycling test, the samples were placed on small crucibles filled to a flat and full level, then subjected to varying temperature points using a temperature-controlled spectrometer. At each temperature point, the samples were held for 1 min before measurement. All measured values were corrected against the light intensity of the control sample, Rhodamine 6G (R6G).

### Characterization of Materials

The structural characterization of the materials was performed using Fourier transform infrared spectroscopy (FTIR; PerkinElmer). Optical properties were evaluated with a photoluminescence spectrometer (HORIBA Jobin Yvon; Fluoromax-4). Morphology and crystal structure were analyzed using a transmission electron microscope (TEM; Hitachi H7100) and an X-ray diffractometer (XRD; Bruker D8 Advance). Additionally, the morphology of FAPbBr_3_@SiO_*x*_@513M was examined using a scanning electron microscope (SEM; Hitachi TM 4000 plus) and a high-resolution transmission electron microscope (HRTEM; JEOL JEM2100F) equipped with EDS mapping (OXFORD Ultim Max TEM).

## Results and Discussion

In the common synthesis of PQDs, OA and OAm are commonly used ligands for dissolving precursors. OA possesses a negatively charged carboxyl functional group, while OAm carries a positively charged amine group. To enhance the effectiveness of the SiO_*x*_ shell coating on the PQDs, this study introduced a small number of siloxane-based APTES as a coupling agent. APTES, functioning as a short-chain ligand with amine groups, competes with OAm for coordination on elements with vacant orbitals during the reaction. To control the photoluminescence, efficiency, and crystallinity of the PQDs, different molar ratios of APTES were tested to determine the optimal addition amount.

Scheme 1 illustrates the surface ligand morphology of FAPbBr_3_-APTES formed in this study, where the blue lines represent OA coordinated to the crystal surface, green indicates OAm, and red lines denote APTES anchoring onto the FAPbBr_3_ surface. Upon exposure to moisture, the siloxy groups of the siloxane undergo a hydrolysis–condensation reaction, facilitating grafting with other added siloxanes. This process then resulted in inorganic SiO_*x*_ coated FAPbBr_3_ PQDs. Following this, organic layer formed by 513M polymers were created to laminate the FAPbBr_3_@SiO_*x*_ materials forming FAPbBr_3_@SiO_*x*_@513M composite. After the formation of the dual-shell structure using 513M polymer, the actual product of FAPbBr_3_@SiO_*x*_@513M composite appearance is displayed in Scheme 1. The final powder state eliminates the need for storage in organic solvents, preventing quantification difficulties and improving feasibility for experimental measurements and commercial application evaluations.

**Scheme 1:**
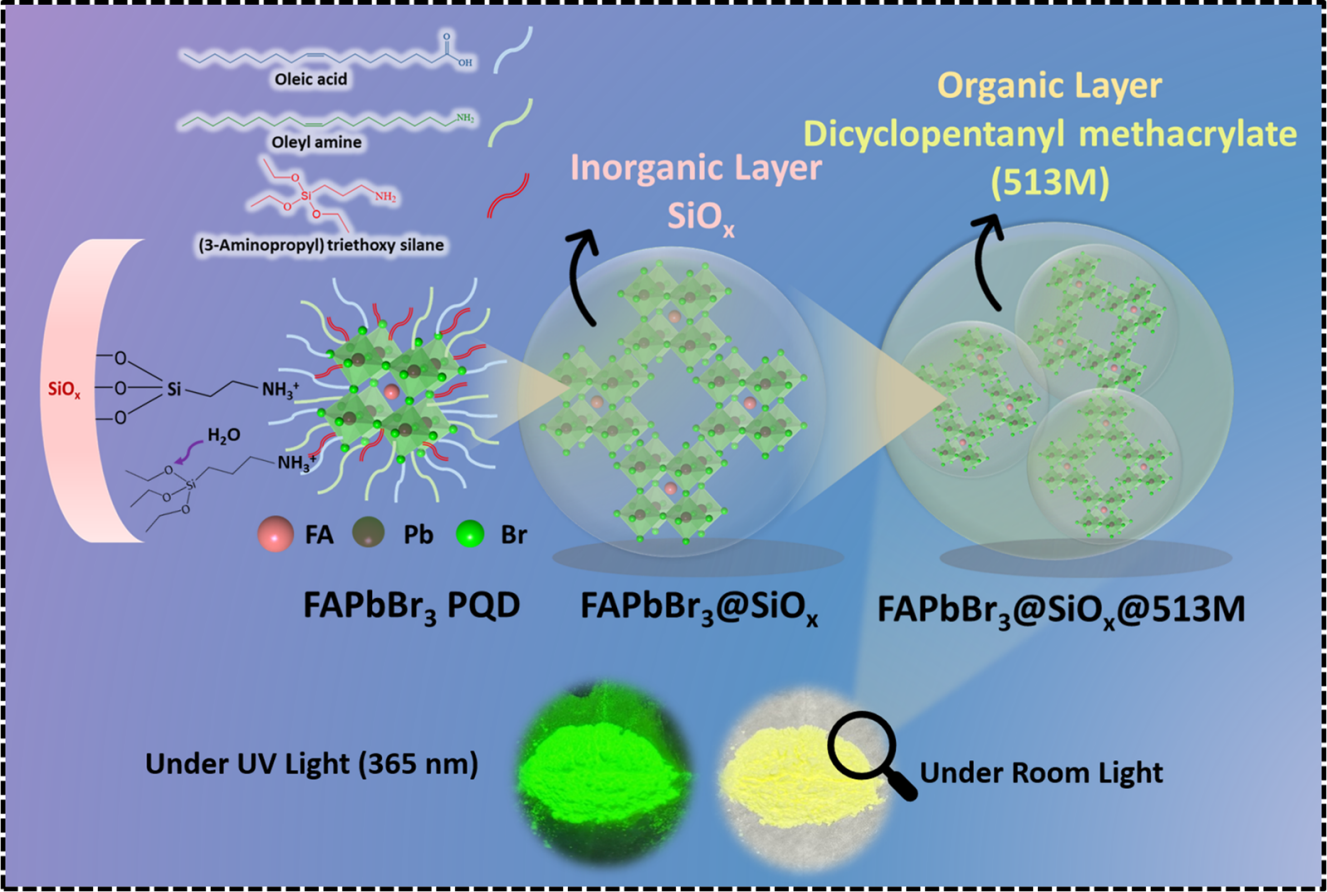
Schematic Representation of Dual Encapsulation for PQDs:FAPbBr_3_@SiO_*x*_@513M Material

The primary focus of this improvement in composite materials remains on maintaining its optical performance, followed by optical stability. FAPbBr_3_ was selected because of its suitable emission range to perform pure green emission light. Various parameters were adjusted, using the equivalent number of APTES as a variable. Measurements were conducted on emission wavelength, fwhm, and PLQY to evaluate the optimal additional amount during the synthesis process. The PLQY of the samples was measured using an integrating sphere coupled to a spectrofluorometer, which allows determination of the ratio of emitted to absorbed photons. Subsequently, encapsulation was carried out to produce FAPbBr_3_@SiO_*x*_ and FAPbBr_3_@SiO_*x*_@513M. The experimental procedure in this study includes both ligands doping and the addition of a coating layer. Based on different stages, the samples were designated as FAPbBr_3_, FAPbBr_3_-APTES, FAPbBr_3_@SiO_*x*_, and FAPbBr_3_@SiO_*x*_@513M. The study examined whether phase changes occurred under different dispersion and stability conditions.

In Figure S1 FAPbBr_3_ PQDs with varying APTES additions of 0, 0.42, 0.85, 1.28, and 1.71 mmol were analyzed against the standard pattern of the crystallography open database (COD-1459033) for the (113) structure, which belongs to the *Pm*-3m space group. The diffraction peaks were observed at 14.8°, 21.0°, 29.8°, 33.5°, 37.9°, 42.8°, and 45.0°, corresponding to the (100), (110), (200), (210), (211), (220), and (300) crystal planes, respectively. All APTES-containing samples exhibited diffraction peaks corresponding to the (113) structure. However, due to preferred orientation effects and sample thickness variations, only the peaks at 14.8°, 29.8°, 33.5°, and 45.5° were clearly detected, while the remaining peaks showed weaker intensities. Additionally, the observed peaks were broader than the standard reference pattern due to the smaller crystallite size.

When the APTES addition increased to 1.28 and 1.71 mmol, non-(113) phase peaks appeared at 12.4° and 37.5°. After applying identical purification parameters, a slight amount of white precipitate was also observed. A comparison with the standard reference pattern of the organic–inorganic perovskite (214) phase FA_2_PbBr_4_ (COD-4003870) ruled out its presence. Further XRD analysis of precursors such as FAAc, PbBr_2_, and APTES revealed that the diffraction peak at 12.4° closely resembled the signal of FAAc. Excessive APTES in the system led to competitive ligand interactions, reducing the OAm/APTES ratio on the PQDs surface.^34^ This accelerated PQDs formation and crystal alignment, potentially replacing A-site vacancies with short-chain positively charged ligands. Under identical reaction conditions, the formation rate of FAPbBr_3_ PQDs increased, product yield decreased, and side products such as precursor microcrystals were generated.

In the FTIR analysis, shown in Figure S2, the spectra of FAPbBr_3_-0 mmol APTES and FAPbBr_3_-0.85 mmol APTES exhibit similar features. Since only a small amount of APTES was added and subsequent purification steps were carried out, no distinct signals corresponding to APTES such as the Si–O bending at 800 cm^–1^, the Si–O–Si stretching at 1090 cm^–1^, or the Si–OH stretching at 3400 cm^–1^ were observed. These characteristic peaks only became evident after the addition of TEOS and the subsequent formation of the SiO_*x*_ layer. In the case of the single-layer coated FAPbBr_3_-0.85 mmol APTES@SiO_*x*_, the spectrum shows overlapping signals from both FAPbBr_3_ and SiO_*x*_. After the second encapsulation, the spectrum of FAPbBr_3_-0.85 mmol APTES@SiO_*x*_@513M closely resembles that of 513M. Nevertheless, the Si–O–Si stretching band around 1090 cm^–1^, partially overlapping with the C–O stretching at 1,160 cm^–1^, remains detectable. A comparison of these data confirms the presence of SiO_*x*_ within the material.

The PL spectra of the aforementioned samples are shown in Figure S3 with the corresponding numerical data listed in Table S1. The results indicate that as the APTES concentration increases, the PL peak gradually redshifts from 518 to 530 nm. Regarding the gradual PL redshift observed with increasing APTES concentration, this behavior is likely influenced by multiple factors. The redshift tendency can be attributed to the formation of surface-related impurities or secondary phases resulting from the excessive addition of APTES. When the APTES concentration surpasses the optimal range, the surplus silane groups may strongly interact with the PQD surface, partially disrupting halide coordination and inducing subtle structural or compositional disorder. These perturbations lead to a slight redshift in the emission peak accompanied by a reduction in overall PL intensity. Below this threshold, the optical properties remain largely stable; however, once impurity formation becomes significant, pronounced optical degradation and a notable decrease in material yield after purification are observed.^3,8,9,35,36^

Moreover, as the APTES concentration increases to 0.85 mmol, the PLQY rises from 90.2 to 96.1%.^35^ However, further addition to 1.28 and 1.71 mmol results in a declining trend. This suggests that an appropriate amount of APTES enhances light conversion efficiency, likely due to its stronger bonding ability, which helps fill A-site surface defects and influences the crystalline arrangement of the PQDs.^36–38^ Based on the result in Figure S4 and Table S2, when no APTES was added, the average lifetime (τ_avg_) of FAPbBr_3_ was 28.90 ns. As the addition increased to 0.42 and 0.85 mmol, τ_avg_ decreased to 19.07 and 19.61 ns, respectively. This reduction was mainly attributed to the optimization of τ_1_ (radiative recombination lifetime), which decreased from 13.66 to 12.52 ns and 12.60 ns. Additionally, the A_1_ proportion changed from 87.28 to 88.83% and 86.37%, indicating that the radiative recombination ratio remained stable, and that the addition of short-chain ligands did not effectively reduce defect states. However, when the APTES addition further increased to 1.28 and 1.71 mmol, τ_avg_ rose to 21.20 and 41.61 ns, respectively. Notably, at 1.71 mmol, τ_1_ significantly increased to 17.93 ns, while the A_1_ proportion dropped to 82.69%, suggesting that not only did the radiative recombination efficiency decline, but the proportion of defect states also increased. This result indicates that excessive APTES affects the crystallization process of PQDs, increasing nonradiative recombination centers and ultimately reducing optical performance.^39,40^ The observed decrease in efficiency with higher APTES concentrations can be attributed to the nature of APTES as a positively charged short-chain ligand. When its concentration increases, it affects the crystallization kinetics of the PQDs by competing with A-site cations for spatial coordination on the [PbBr_6_]^4–^ surface. As ligand occupancy at the surface terminates the reaction, unreacted precursors remain, leading to the formation of additional impurity crystals. This phenomenon reduces the overall yield of FAPbBr_3_ and increases impurity levels. Without effective purification, the PL intensity, efficiency, and even emission wavelength of the samples may be adversely affected. Based on these observations, the critical APTES addition in this experiment was determined to be 0.85 mmol, yielding phase-pure FAPbBr_3_-0.85 mmol APTES samples with APTES ligands successfully incorporated on the PQDs surface. This formulation facilitates the subsequent formation of the SiO_*x*_ coating layer on FAPbBr_3_ PQDs.

Furthermore, we continued the materials analysis by conducting a comparative analysis of the XRD patterns of FAPbBr_3_, FAPbBr_3_@SiO_*x*_, and FAPbBr_3_@SiO_*x*_@513M. In Figure 1a, it was observed that the sample FAPbBr_3_-0.85 mmol APTES@SiO_*x*_ exhibited an amorphous SiO_*x*_ signal in the 17° to 25° range, which matched the reference SiO_*x*_ sample.^39^ Since the X-rays could still penetrate through the single coating layer, the FAPbBr_3_ crystal signals remained observable. In contrast, after undergoing free radical polymerization to form a polymer coating layer, the sample FAPbBr_3_-0.85 mmol APTES@SiO_*x*_@513 M exhibited an amorphous phase peak in the 12–22° range, aligning with the reference 513 M sample. Due to the increased coating thickness, the FAPbBr_3_ crystal signals became significantly weaker and were difficult to detect in the final powder product.

**Figure 1:**
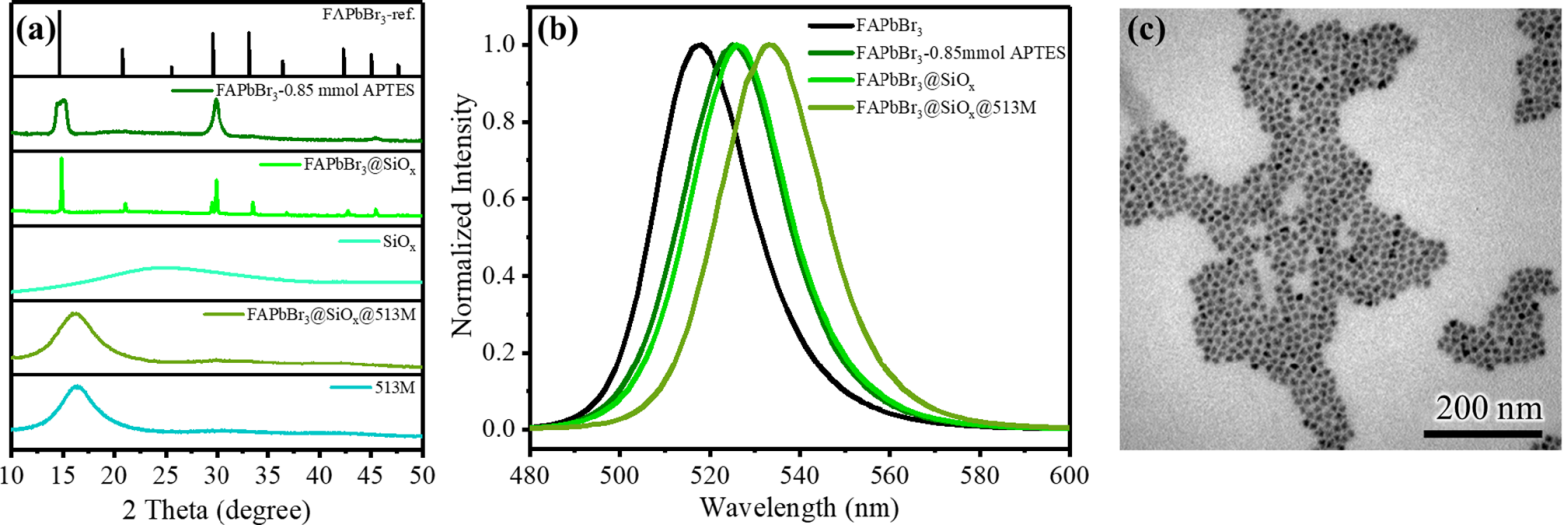
(a) X-ray diffraction patterns of FAPbBr_3_ reference (COD-1459033), FAPbBr_3_-0.85 mmol APTES, FAPbBr_3_@SiO_*x*_, SiO_*x*_, FAPbBr_3_@SiO_*x*_@513M, and 513M. (b) PL emission spectra of the materials. (c) TEM image of traditional FAPbBr_3_ PQDs where the ligands consist only of OA and OAm.

As additional coating layers were applied to PQDs, the emission peak exhibits varying degrees of redshift in Figure 1b. This phenomenon was primarily attributed to the effect of self-absorption. This effect occurs when fluorescent materials with the same carrier are present, where fluorescence emitted by one unit can be reabsorbed by adjacent units depending on concentration, absorption, and emission wavelengths, leading to re-emission at a lower energy and red-shifted wavelength.^41^ In the coated materials, the volume of the SiO_*x*_ and 513 M layers, as well as the internal material distribution density, contributed to different degrees of self-absorption, ultimately causing the observed redshift in emission. Additionally, as the number of coating layers increases, the PLQY followed a decreasing trend, as shown in Table S3. Factors such as surface irregularities, light transmittance, and coating thickness contributed to this decline. Compared to the PLQY of 96.1% for FAPbBr_3_-0.85 mmol APTES, the PLQY of FAPbBr_3_@SiO_*x*_ and FAPbBr_3_@SiO_*x*_@513 M decreases to 95.2 and 52.3%, respectively, caused by the aforementioned effects.

The PLQY of the SiO_*x*_-encapsulated PQDs was found to be slightly lower than that of the pristine samples, which can be attributed to the partial reabsorption of emitted photons within the encapsulation layer.^40,42^ The double-layer structure, while primarily designed for stability enhancement, also affects the optical path length and local dielectric environment.^16,30,43^ The polymer shell exhibits high optical transparency and a moderate refractive index, which helps to maintain efficient light extraction and minimizes the loss of emission intensity.^28^ The absorption cross-section and color conversion efficiency are influenced by both the optical clarity and thickness of the encapsulation layer.^3,6^ Excessive coating thickness may lead to scattering and self-absorption, whereas an overly thin layer compromises protection against moisture and heat.^16,30^ In this study, encapsulation thickness was optimized (FAPbBr_3_@SiO_*x*_ suspension and 513 M ratio = 1 mL: 0.6 g) to achieve a balance between high transparency and robust environmental stability.

The morphology of the synthesized FAPbBr_3_ with no APTES is shown in Figure 1c, where most particles can be identified as monodispersed as cubic. The particles are monodispersed and arranged regularly, with majority sizes ranging between 9 and 13 nm.

To further investigate the influence of APTES on the encapsulation behavior and morphology of FAPbBr_3_, TEM and HRTEM analyses were conducted. Comparing Figure 2a FAPbBr_3_-0 mmol APTES@SiO_*x*_ and Figure 2b FAPbBr_3_-0.85 mmol APTES@SiO_*x*_, the dark dotted structures represent FAPbBr_3_, while the irregular light gray structures correspond to SiO_*x*_. In the case of FAPbBr_3_-0 mmol APTES@SiO_*x*_, most FAPbBr_3_ particles remain unencapsulated by SiO_*x*_, indicating a more random encapsulation selectivity. In contrast, FAPbBr_3_-0.85 mmol APTES@SiO_*x*_ shows a higher proportion of FAPbBr_3_ encapsulated by SiO_*x*_. A comparison of the TEM images clearly indicates that the addition of APTES enhances the coupling ability, resulting in a more uniform and complete encapsulation of FAPbBr_3_ by SiO_*x*_. Additionally, the encapsulated FAPbBr_3_ PQDs particles demonstrate enhanced stability under high-voltage electron beam exposure. This allows for HRTEM analysis of the detailed morphology of a single encapsulated FAPbBr_3_@SiO_*x*_ particle in Figure S5a,b presents a high-magnification image focusing on the FAPbBr_3_ PQDs, where the lattice structure and spacing, calculated to be approximately 0.3 nm, correspond to the (200) plane observed in XRD analysis.^44–46^ Figure S5c displays multiple encapsulated FAPbBr_3_@SiO_*x*_ particles, which dominate the synthesis results. In Figure S5d, the lattice structure of encapsulated FAPbBr_3_ is still observable, confirming that most quantum dots retain their crystallinity after the hydrolysis–condensation reaction. Their unidirectional and orderly alignment further verifies the single-crystal nature of the material.

**Figure 2:**
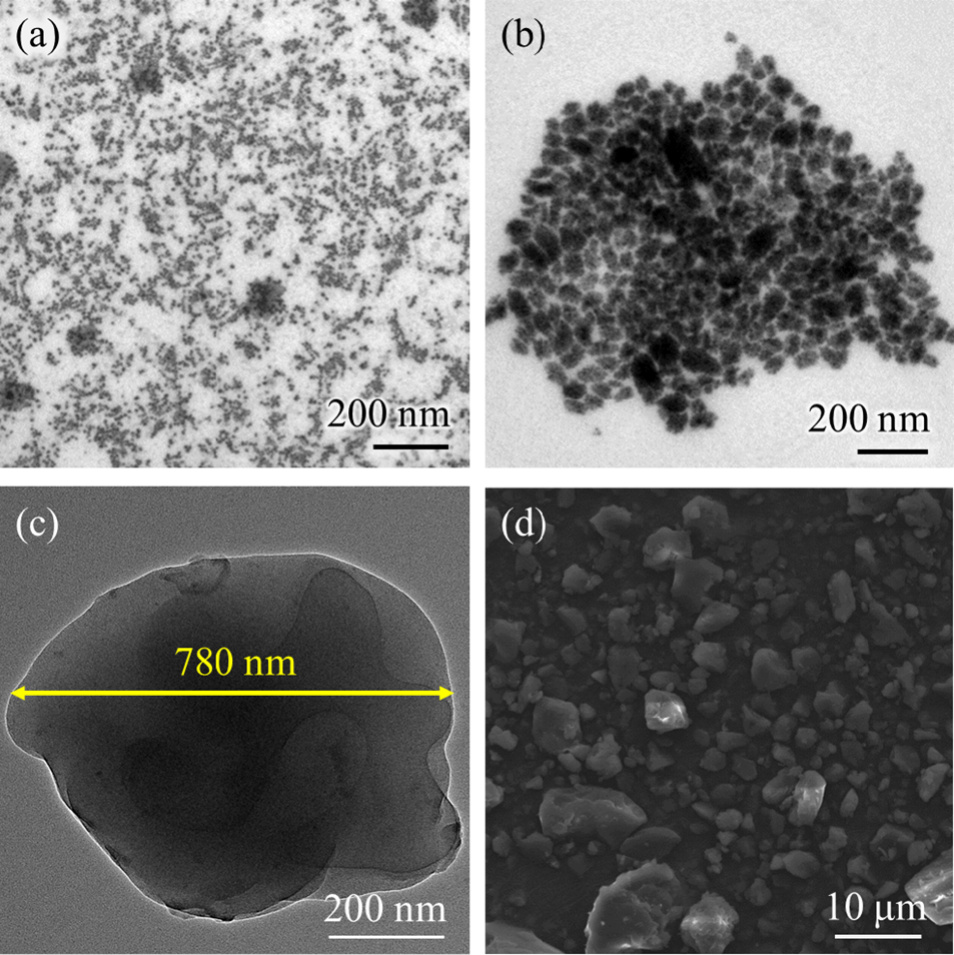
(a) TEM image of FAPbBr_3_@SiO_*x*_ with no APTES added. (b) TEM image of FAPbBr_3_-0.85 mmol APTES@SiO_*x*_. (c) TEM image of FAPbBr_3_@SiO_*x*_@513M. (d) SEM image of FAPbBr_3_@SiO_*x*_@513M.

After secondary granulation, FAPbBr_3_@SiO_*x*_@513 M particles mostly exceed 200 nm in size. The polymer coating layer, composed of dicyclopentanyl methacrylate is highly transparent, and its thickness is significantly reduced after crushing. This property allows TEM imaging to still provide insights into the internal distribution of FAPbBr_3_@SiO_*x*_ and elemental composition. Figure 2c shows the TEM results, where the outermost polymer layer, composed of carbon, hydrogen, oxygen, and phosphorus, appears as a lighter region. Ideally, these particles are uniformly dispersed, minimizing self-absorption effects. Elemental mapping in Figure S6 reveals a high density of carbon signals in light-colored areas. Due to the similar atomic numbers of silicon, oxygen, carbon, hydrogen, and phosphorus, distinguishing them is challenging. However, the overlapping distributions of silicon with Pb and Br suggest that FAPbBr_3_ is predominantly encapsulated by SiO_*x*_ before being further coated by 513M, improving its environmental stability. Figure S6 also presents the EDS elemental composition of FAPbBr_3_@SiO_*x*_@513M, showing that C accounts for nearly 90%, Si around 3%, and Pb and Br approximately 1.2% each. Figure 2d displays images obtained using the Secondary Electron Detector (SED) mode, revealing particle sizes ranging from 0.8 to 20 nm. Figure S7 shows the EDS elemental mapping results, indicating surface C content at 61.67% and O at 32.66%, primarily attributed to the outermost coating layer. The detected Si content is 3.71%, originating from the first coating layer and exposed regions after granulation procedure. Pb and Br contents in FAPbBr_3_ are 0% and 0.18%, respectively.

In the reliability test section, FAPbBr_3_, FAPbBr_3_@SiO_*x*_, and FAPbBr_3_@SiO_*x*_@513 M were subjected to at least 336 h in a commercial adhesive containing methacrylic acid. These tests aimed to assess the effectiveness of single-layer and double-layer coatings in protecting the core FAPbBr_3_ PQDs from quenching factors and ensuring long-term stability under challenging conditions. Additionally, FAPbBr_3_@SiO_*x*_@513M was tested for water resistance and underwent temperature-dependent spectroscopic analysis to identify changes in material behavior under various external factors. The results of the light and heat resistance tests are shown in Figure 3a,b, respectively. Under continuous blue light exposure, FAPbBr_3_ exhibited significant degradation, with its intensity dropping below 5% of the initial value after 96 h and reaching complete quenching by 336 h. FAPbBr_3_@SiO_*x*_ performed slightly better, retaining 40% of its initial intensity at 120 h and fully quenching at 336 h. In contrast, FAPbBr_3_@SiO_*x*_@513M demonstrated superior stability, maintaining 74.4% of its initial intensity at 336 h and linearly declining to 58.38% at 504 h. The primary degradation mechanism under light exposure is the generation of free radicals, where electrons from PQDs react with O_2_ and CO_2_, producing free radicals that interact with organic A-site cations, leading to the release of volatile gases such as CH_3_NH_2_, C_2_H_5_NH_3_, and CH_4_N_2_, causing irreversible crystal lattice degradation.^9,47,48^ In the heat resistance test conducted at 60 °C, FAPbBr_3_ showed a slower decline in intensity compared to light exposure, maintaining more than 5% of its initial intensity until 168 h but fully quenching by 336 h, indicating that light posed a more severe challenge. FAPbBr_3_@SiO_*x*_ retained 20% of its intensity at 168 h and 5% at 336 h. FAPbBr_3_@SiO_*x*_@513M again outperformed the others, experiencing noticeable quenching within the first 24 h but maintaining a relatively stable decline, thereafter, retaining 65.1% of its intensity at 336 h and 71.05% at 504 h.

**Figure 3:**
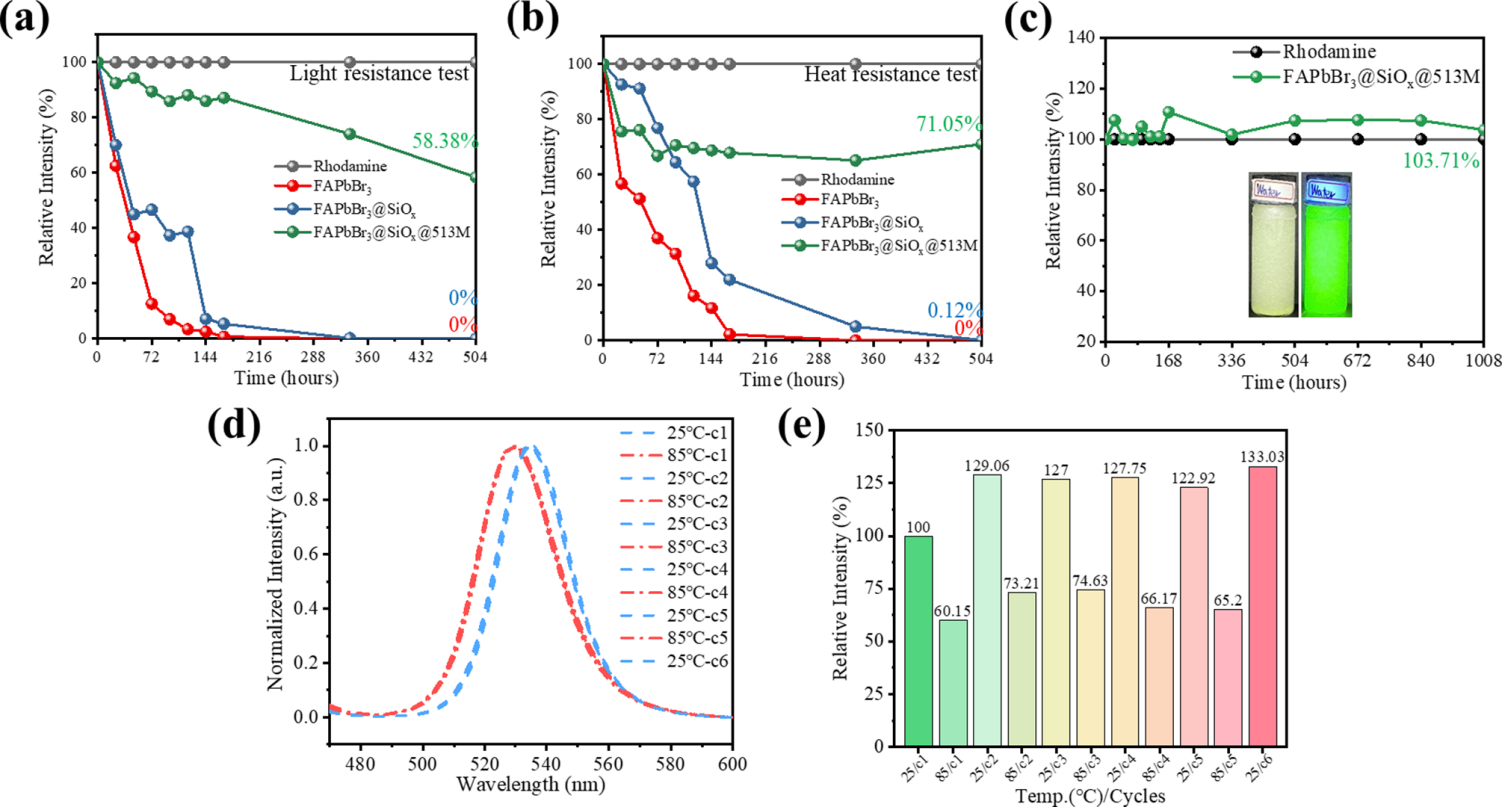
(a,b) Comparison of light resistance and heat resistance tests for the samples after 336 h, with the black line representing the standard reference (R6G), the red line representing the intensity trend of FAPbBr_3_, the blue line representing FAPbBr_3_@SiO_*x*_, and the green line representing FAPbBr_3_@SiO_*x*_@513M. (c) Trend line illustrates the light intensity of FAPbBr_3_@SiO_*x*_@513M underwater environment over time. (d) Bar chart showing the spectral intensity of FAPbBr_3_@SiO_*x*_@513M during thermal cycling between 25 and 85 °C. (e) Normalized parameters from (d) highlighting changes in the material’s main emission peak and fwhm over the thermal cycles.

The water resistance test results for FAPbBr_3_@SiO_*x*_@513M are shown in Figure 3c. After 1008 h, the material retained its full light intensity. This result can be attributed to the hydrophobic nature of the secondary encapsulation layer, 513M, which prevents water and oxygen from causing surface ligand detachment or directly reacting with the surface crystals. This advantage effectively inhibits Ostwald ripening and surface trap states, thereby avoiding quenching phenomena.^4^ The minor fluctuations observed are primarily associated with the intrinsic sensitivity of PQDs to external stimuli, such as light exposure, local environmental variations, and measurement conditions that commonly happened in perovskite-based nanomaterials. Moreover, Figure 3c monitors the time-dependent photoluminescence response of the same FAPbBr_3_@SiO_*x*_@513M sample under an underwater environment, which introduces additional factors such as refractive-index mismatch, light scattering, and transient interfacial interactions with water molecules. These effects can lead to short-term intensity fluctuations without causing irreversible degradation of the perovskite core. Importantly, the observed fluctuations are not significant and do not fall below 100% of the initial normalized value, indicating that no irreversible degradation or performance loss occurs during the test.

A comparative summary of reported double-layer SiO_2_/polymer encapsulation strategies for CsPbBr_3_, FAPbBr_3_, and related perovskites, along with their stability toward light, heat, and moisture, is provided in Table S4. Double-layer encapsulation employing an inorganic SiO_2_ inner layer and an outer polymer shell has been widely reported to enhance the stability of CsPbBr_3_-based perovskites. For instance, CsPbBr_3_@SiO_2_@PS exhibited >90% retention under prolonged UV irradiation, heating at 85 °C, and exposure to 85% relative humidity, while CsPbBr_3_@Cs_4_PbBr_6_/SiO_2_/PDMS maintained >98% of its luminescence in 50% humidity for two months. Such results highlight the synergistic protection afforded by inorganic–organic bilayers, in which SiO_2_ restricts oxygen and moisture diffusion, whereas the polymer serves as a flexible hydrophobic barrier. Moreover, specialized polymers such as 513 M have shown remarkable water resistance, as demonstrated by FAPbBr_3_@SiO_*x*_@513M, which retained 71.05% stability after 3 weeks at 60 °C and even improved luminescence (>100%) after immersion in water for one month. However, compared to CsPbBr_3_, FAPbBr_3_ remains intrinsically less stable under heat, light, and humidity, and reports on double-layer encapsulation for FAPbBr_3_ are relatively scarce and often lack comprehensive stability data. For example, FAPbBr_3_/SiO_2_ was reported without quantitative durability, while FAPbBr_3_–PLLA retained only ∼20% after one month of water immersion. These findings suggest that although SiO_2_/polymer double-layer encapsulation is well established for CsPbBr_3_, further optimization and systematic evaluation are still required for FAPbBr_3_ to achieve comparable stability.

The variable-temperature PL intensity test shown in Figure 3d,e represent experiment involved a 25 °C/85 °C thermal cycling test. In Figure 3d, blue shifts in the emission peak occurred upon heating but returned to the original wavelength when the temperature decreased to room temperature.^47,49^ Figure 3e indicates that after the first thermal cycle, the luminescence intensity increased to over 120% of the initial value and remained at this level in subsequent cycles. This enhancement is attributed to the thermal annealing effect, which improves the luminescence intensity of the PQDs, and the softening of the polymer encapsulation layer upon reaching its glass transition temperature (*T*_g_). This softening reduces surface irregularities and light scattering, ultimately improving bidirectional light flux.^50,51^

Regarding encapsulation yield and batch-to-batch reproducibility, because the polymer matrix represents >95% of the final composite mass, the total output weight provides a reliable indirect measure of encapsulation yield. Across repeated batches prepared under identical conditions, the variation in total composite mass is consistently within ±1–2%, indicating good reproducibility of the overall fabrication process. The workflow intentionally uses slight excess volumes to compensate for minor losses (e.g., floating particulates) and ensure complete filling of test units, minimizing batch-to-batch fluctuation. While the absolute mass of the perovskite component cannot be accurately weighed due to its extremely small quantity and adherence to container surfaces, the constant PQD-to-polymer mixing ratio and the low (<2%) variation in final mass strongly suggest that the encapsulation process exhibits stable and scalable performance suitable for industrial optimization.

Moreover, the scalability of this material’s production, the full encapsulation strategy used in this work is compatible with standard batch-type industrial synthesis. A typical commercial batch process yields approximately 100 g of solid product. Based on established industrial practices and previously reported patent-scale QDs encapsulation processes, the reaction conditions demonstrated in this study (precursor ratios, moisture-controlled SiO_*x*_ condensation, and polymer curing conditions) can be proportionally scaled without altering the core chemistry. This suggests that our laboratory-scale procedures can be translated into industrially relevant production volumes with predictable reproducibility.

A simulated light emitting diode (LED) was designed by using a 14 × 27 mil blue light chip as the excitation source. As shown in the Figure 4a, the surface of the chip was coated with a mixed colloid of FAPbBr_3_@SiO_*x*_@513M and connected to a power supply, with the voltage set to 3 V. Currents of 20, 30, 40, 50, 60, 70, 80, 90, and 100 mA were applied sequentially to evaluate its luminescent performance. As depicted in Figure 4a,b, the luminous intensity gradually increased between 20 and 60 mA, reaching its peak at 60 mA. However, when the current was further increased to 100 mA, the intensity of green light decreased progressively, falling to 83.6% of its maximum intensity at 100 mA. At this point, the green light conversion efficiency was 82.76%. The spectral analysis reveals that after reaching the peak at 60 mA, the green light emission began to decline, while the blue light peak intensity increased. This pattern is attributable not only to the natural rise in blue light intensity with increased current but also to the degradation of the perovskite material due to poor photostability, leading to reduced blue light absorption and green light quenching.

**Figure 4:**
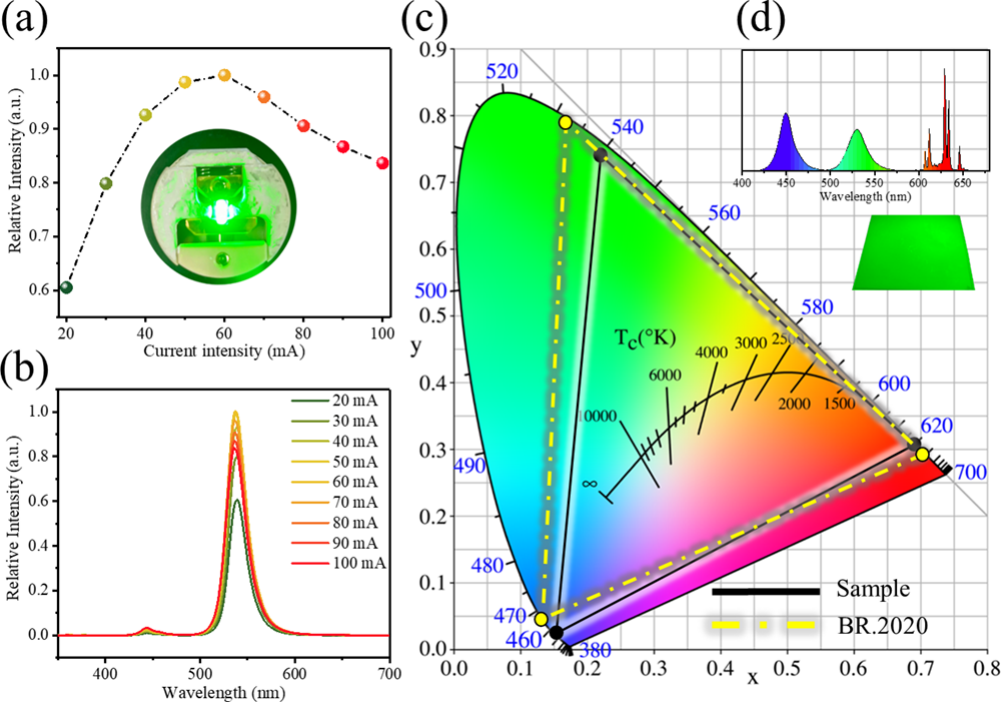
Simulated LED using FAPbBr_3_@SiO_*x*_@513M for (a) the trend of input current versus luminous intensity; (b) PL spectra under different current conditions; (c) color gamut coverage of the simulated optical film on a chromaticity diagram, incorporating a GaN blue light chip, FAPbBr_3_@SiO_*x*_@513M optical film, and KSF phosphor (K_2_SiF_6_:Mn^4+^); and (d) full visible light spectrum of (c).

The optical thin film simulated in this research offers numerous advantages, including low-cost deposition techniques, minimal material consumption, and excellent optical properties. The optical thin film in Figure 4 was prepared by spin-coating or blade-coating a thin layer of a commercial organic polymer onto a transparent PET substrate, followed by precise layer alignment using molding tools. The assembled film was then UV-cured under an inert atmosphere to ensure strong interfacial bonding and long-term environmental durability. These characteristics position PQDs thin films as a promising candidate for emerging optoelectronic technologies, despite being in the developmental phase and not yet ready for commercial applications. The fabricated FAPbBr_3_@SiO_*x*_@513M color-converting optical film, as demonstrated in Figure 4c, holds potential for high-end display technologies. Leveraging the exceptional PL properties of PQDs, this film achieves high PLQY, superior color purity, and tunable emission wavelengths. In Figure 4d, the emission wavelength of the FAPbBr_3_@SiO_*x*_@513M film was centered at 532 nm. When combined with a GaN blue light chip and KSF phosphor (K_2_SiF_6_:Mn^4+^), the resulting visible spectrum was measured. The corresponding color coordinates on the color gamut diagram were (0.1485, 0.0422) for blue, (0.2462, 0.7134) for red, and (0.6928, 0.3070) for green, achieving a BT.2020 color gamut coverage of 79.63%. Overcoming challenges related to stability and scalability in manufacturing will pave the way for a groundbreaking milestone in the next generation display technologies.

From a cost perspective, the combined SiO_*x*_ and 513M encapsulation significantly enhances environmental stability, which directly affects material consumption and overall QD-film manufacturing cost. Commercial QD films typically employ a PET/barrier/QD-polymer/barrier/PET multilayer structure. Within this architecture, two primary cost-reduction mechanisms become relevant: (1) The high PLQY and improved stability of SiO_*x*_-coated FAPbBr_3_ (either alone or embedded in 513M) allow the required QD loading in the coated composite to be reduced without sacrificing optical conversion efficiency. This directly decreases the amount of perovskite QD material needed per film. (2) Enhanced moisture and oxygen resistance from the dual encapsulation system (SiO_*x*_ + 513M) lowers the performance requirements of the external barrier films. Because barrier-film cost scales strongly with water-vapor/oxygen transmission rate (WVTR) specifications, the ability to use lower-specification barrier coatings results in a substantial reduction of total material cost.

As shown in Table S3, the PLQYs of FAPbBr_3_@SiO_*x*_ and FAPbBr_3_@SiO_*x*_@513M are 95.2 and 52.3%, respectively. SiO_*x*_ encapsulation alone mainly contributes to mechanism (1) and partially to (2), whereas the combined SiO_*x*_ + 513M encapsulation more effectively enables mechanism (2). In a forward-looking scenario, where the encapsulation sufficiently mitigates water-oxygen-induced degradation, the outer barrier-coating layer may become unnecessary, potentially eliminating a major cost component associated with the PET/barrier structure.

Additionally, a representative material-cost distribution for commercial QD films is provided in Figure S8, where the QD material, barrier films, and coating/lamination account for approximately 45, 42, and 8% of total cost, respectively. The improved encapsulated material used in this work reduces costs in two ways. First, enhanced optical stability enables a lower QD loading while maintaining equivalent color-conversion efficiency, thereby decreasing the most expensive component of the film stack. Second, the improved moisture and oxygen resistance at the particle level relaxes the WVTR requirement of the barrier layers (from ∼10^–3^ to ∼10^–2^ g m^–2^ day^–2^), which significantly lowers the cost of the barrier films and, in certain cases, may eliminate the need for an additional barrier-coating layer. These effects highlight the scalability and cost-effectiveness of our encapsulation approach for industrial implementation.

## Conclusions

In this research, a coating strategy was employed to enhance the stability of FAPbBr_3_ PQDs. SiO_*x*_ was chosen as the first coating layer, preserving the excellent optical properties of the material. Additionally, the surface of FAPbBr_3_ was modified with APTES as a coupling agent to improve both the material’s stability and the efficiency and precision of the SiO_*x*_ coating. After the first coating layer, FAPbBr_3_@SiO_*x*_ demonstrated improvements in light and heat tolerance, making it suitable for further processing. To further enhance its resistance to light, heat, water, and oxygen, 513M hydrophobic polymer was selected as the second coating layer. Tolerance tests revealed significant improvements in stability. Ultimately, the organic–inorganic double-coated PQDs material, FAPbBr_3_@SiO_*x*_@513M, was successfully synthesized. This material can be preliminarily fabricated into end-use products, such as LEDs and optical films, showcasing its potential for practical applications.

## Supplementary Material



## Notes

Detailed information about the optical properties of the materials (e.g., PL and TRPL spectra), XRD pattern, HRTEM images, and EDS elemental distributions of the prepared materials (PDF)
