# Supplementary material for: Inorganic–Organic Multicoating Layer Encapsulation of Formamidine Lead Halide Perovskite Quantum Dots for Lighting Applications
Source: ACS Appl Mater Interfaces. 2026 Feb 11;18(7):11912–22. doi: 10.1021/acsami.5c24129 (PMC12954669; doi:10.1021/acsami.5c24129)
Supplement: Supplementary file 1 [file am5c24129_si_001.pdf]

Supporting Information

Inorganic-Organic Multicoating Layer Encapsulation of Formamidine Lead Halide Perovskite Quantum Dots for Lighting Applications

Ling Hsuan Chung[a,†], Andi Magattang Gafur Muchlis[a,†], Po-Chun Li[a], Yan Chung Lai[a], Yuan-Hong Chen[a], Jung-An Cheng[c], and Chun Che Lin[a,b,*]

[a] Institute of Organic and Polymeric Materials, National Taipei University of Technology, Taipei, 106, Taiwan.

[b] Research and Development Center for Smart Textile Technology, National Taipei University of Technology, Taipei, 106, Taiwan.

[c] Foxconn Technology Co., Ltd., Miaoli County, 35053, Taiwan.

† These authors contributed equally to this work.

* Corresponding author, E-mail: cclin0530@mail.ntut.edu.tw (C. C. Lin).

Table S1. The spectral values of $FAPbBr_3$-0 mmol APTES, $FAPbBr_3$-0.42 mmol APTES, $FAPbBr_3$-0.85 mmol APTES, $FAPbBr_3$-1.28 mmol APTES and $FAPbBr_3$-1.71 mmol APTES at an absorbance of 0.1.

Sample	Main emission (nm)	FWHM (nm)	PLQY (%)
$FAPbBr_3$-0 mmol APTES	518	25.2	90.2
$FAPbBr_3$-0.42 mmol APTES	520	26.8	89.7
$FAPbBr_3$-0.85 mmol APTES	525	26.5	96.1
$FAPbBr_3$-1.28 mmol APTES	527	24.1	86.9
$FAPbBr_3$-1.71 mmol APTES	530	24.2	87.5

Table S2. The fitted fluorescence lifetime values of $FAPbBr_3$-0 mmol APTES, $FAPbBr_3$-0.42 mmol APTES, $FAPbBr_3$-0.85 mmol APTES, $FAPbBr_3$-1.28 mmol APTES and $FAPbBr_3$-1.71 mmol APTES at an absorbance of 0.1.

Sample	A_1	τ_1 (ns)	A_2	τ_2 (ns)	τ_{avg} (ns)
$FAPbBr_3$-0 mmol APTES	5931.252	13.66416	864.3195	54.91607	28.90
$FAPbBr_3$-0.42 mmol APTES	5834.747	12.51957	733.8679	36.80305	19.07
$FAPbBr_3$-0.85 mmol APTES	5676.615	12.60081	895.5913	35.41605	19.61
$FAPbBr_3$-1.28 mmol APTES	5693.822	12.60963	917.7795	38.59686	21.20
$FAPbBr_3$-1.71 mmol APTES	5887.484	17.93943	1232.77	70.41392	41.61

Table S3. The spectral values of $FAPbBr_3$-0 mmol APTES, $FAPbBr_3$-0.85 mmol APTES, $FAPbBr_3@SiO_x$, and $FAPbBr_3@SiO_x@$513M.

Sample	Main emission (nm)	FWHM (nm)	PLQY (%)
$FAPbBr_3$-0 mmol APTES	518	25.2	90.2
$FAPbBr_3$-0.85 mmol APTES	525	26.5	96.1
$FAPbBr_3@SiO_x$	526	25.4	95.2
$FAPbBr_3@SiO_x@$513M	532	27.0	52.3

Table S4. Summary of double-layer SiO_x/polymer encapsulation strategies for related perovskites, along with their stability toward light, heat, and moisture.

Material	Stability toward UV light	Stability toward temperature	Stability toward water/humidity	Ref.
$FAPbBr_3$@SiO_x@513M<br>Perovskite: $FAPbBr_3$<br>1st Coating: SiO_x<br>2nd Coating: 513M	58.38% after being irradiated for 3 weeks.	71.05% after heating at 60°C for 3 weeks.	103.71% after immersed in water for more than 1 month.	This work
$CsPbBr_3$@SiO_2@PS<br>Perovskite: $CsPbBr_3$<br>1st Coating: SiO_2<br>2nd Coating: Polystyrene	>90% after being irradiated for 1 month.	>90% after heating at 85°C.	>90% after putting in 85% humidity.	1
CPB@S@P<br>Perovskite: Zn-doped $CsPbBr_3$<br>1st Coating: Silica<br>2nd Coating: PVP and NIPAM polymers	Not specified	<40% after heating for 60 min.	Not specified	2
$MAPbBr_3$@SiO_2/PVDF<br>Perovskite: $MAPbBr_3$<br>1st Coating: SiO_2<br>2nd Coating: PVDF	~83% after being irradiated for 50 hours.	Not specified	Not specified	3
$CsPbBr_3$@Cs_4PbBr_6/SiO_2/PDMS<br>Perovskite: $CsPbBr_3$@Cs_4PbBr_6<br>1st Coating: SiO_2<br>2nd Coating: PDMS	Not specified	~50% after heating at 70°C.	>98% after put in 50% humidity for 2 months.	4
PZ@513M@SiO_2<br>Perovskite: $CsPbBr_3$@ZIF-8<br>1st Coating: 513M<br>2nd Coating: SiO_2	N/A	N/A	Stable (100%) in water for 5 minutes.	5
$FAPbBr_3$/SiO_2<br>Perovskite: $FAPbBr_3$<br>Coating: SiO_2	N/A	N/A	N/A	6
$FAPbBr_3$-PLLA<br>Perovskite: $FAPbBr_3$<br>Coating: Polylactic acid	N/A	N/A	~20% after immersed in water for 1 month.	7

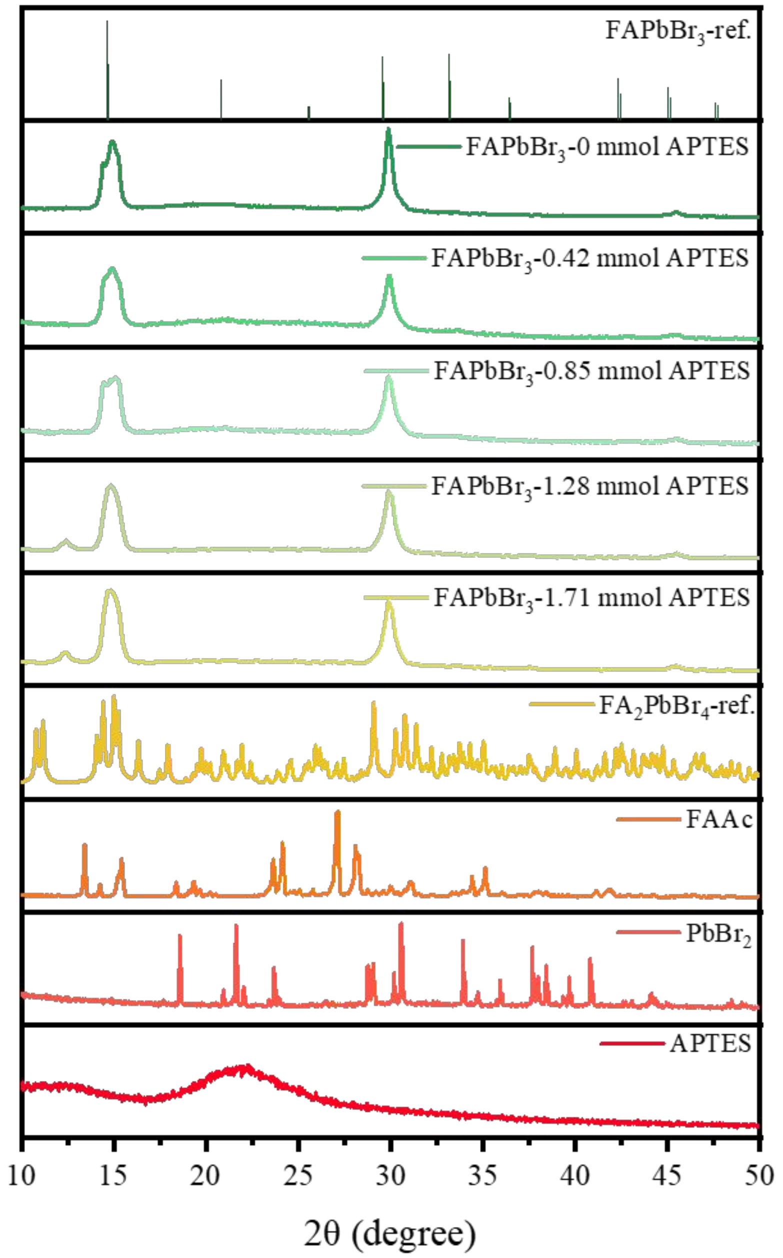

Figure S1. From top to bottom, the XRD patterns correspond to the standard spectrum of $FAPbBr_3$, $FAPbBr_3$-0 mmol APTES, $FAPbBr_3$-0.42 mmol APTES, $FAPbBr_3$-0.85 mmol APTES, $FAPbBr_3$-1.28 mmol APTES, $FAPbBr_3$-1.71 mmol APTES, the standard spectrum of FA_2PbBr_4, FAAc, $PbBr_2$, and APTES.

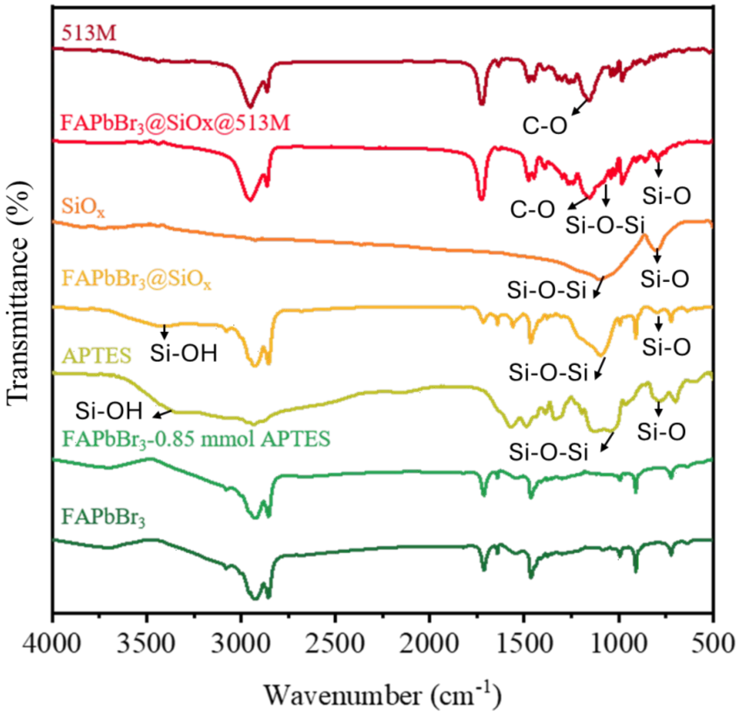

Figure S2. From top to bottom, the FTIR patterns correspond to the standard spectrum of 513M, $FAPbBr_3@SiO_x@513M$, SiO_x, $FAPbBr_3@SiO_x$, APTES, $FAPbBr_3$-0.85 mmol APTES, and $FAPbBr_3$.

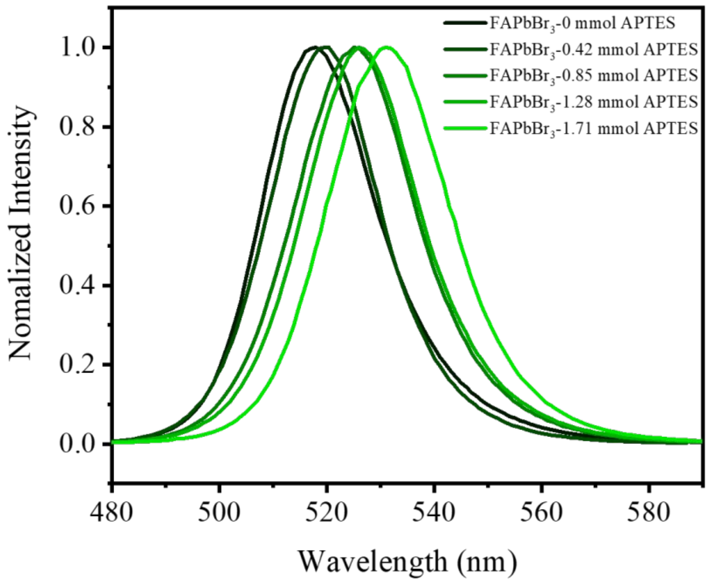

Figure S3. The photoluminescence spectra of $FAPbBr_3$-0 mmol APTES, $FAPbBr_3$-0.42 mmol APTES, $FAPbBr_3$-0.85 mmol APTES, $FAPbBr_3$-1.28 mmol APTES, $FAPbBr_3$-1.71 mmol APTES at a fixed absorbance of 0.1.

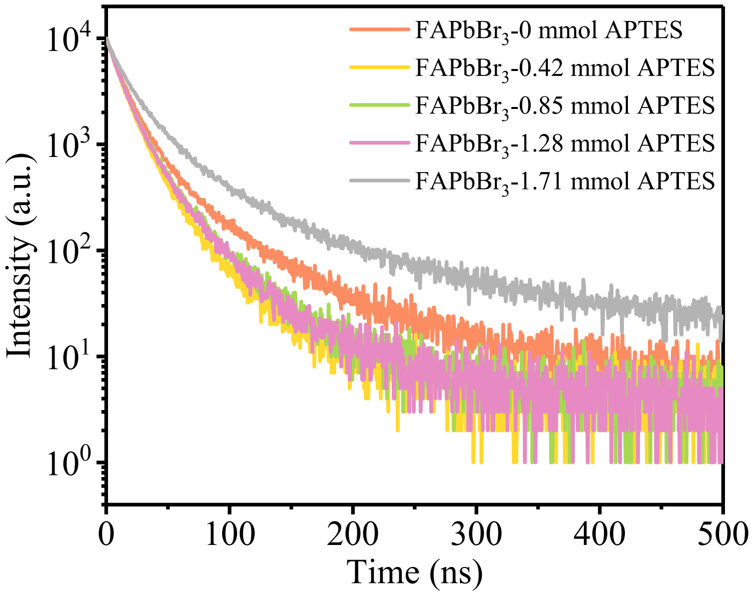

Figure S4. The photoluminescence decay of $FAPbBr_3$-0 mmol APTES, $FAPbBr_3$-0.42 mmol APTES, $FAPbBr_3$-0.85 mmol APTES, $FAPbBr_3$-1.28 mmol APTES, $FAPbBr_3$-1.71 mmol APTES at a fixed absorbance of 0.1.

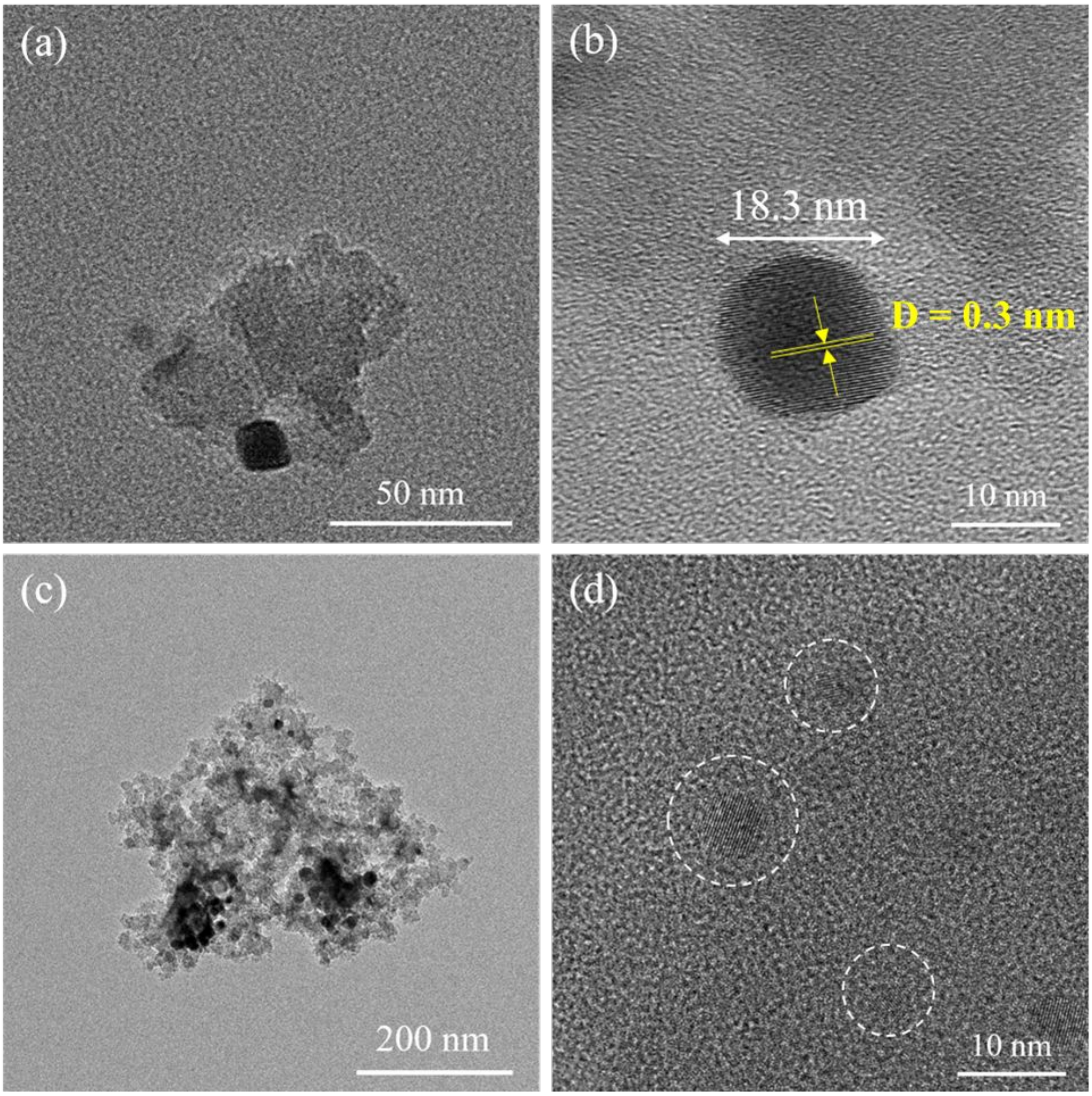

Figure S5. HRTEM (JEM-2100F) images of the $FAPbBr_3@SiO_x$ sample: (a) a single quantum dot encapsulated as $FAPbBr_3@SiO_x$ particle, (b) a high-magnification image of (a), showing the lattice arrangement of $FAPbBr_3$, (c) multiple encapsulated $FAPbBr_3@SiO_x$ particles, and (d) a high-magnification image of (c), revealing the lattice structure of $FAPbBr_3$.

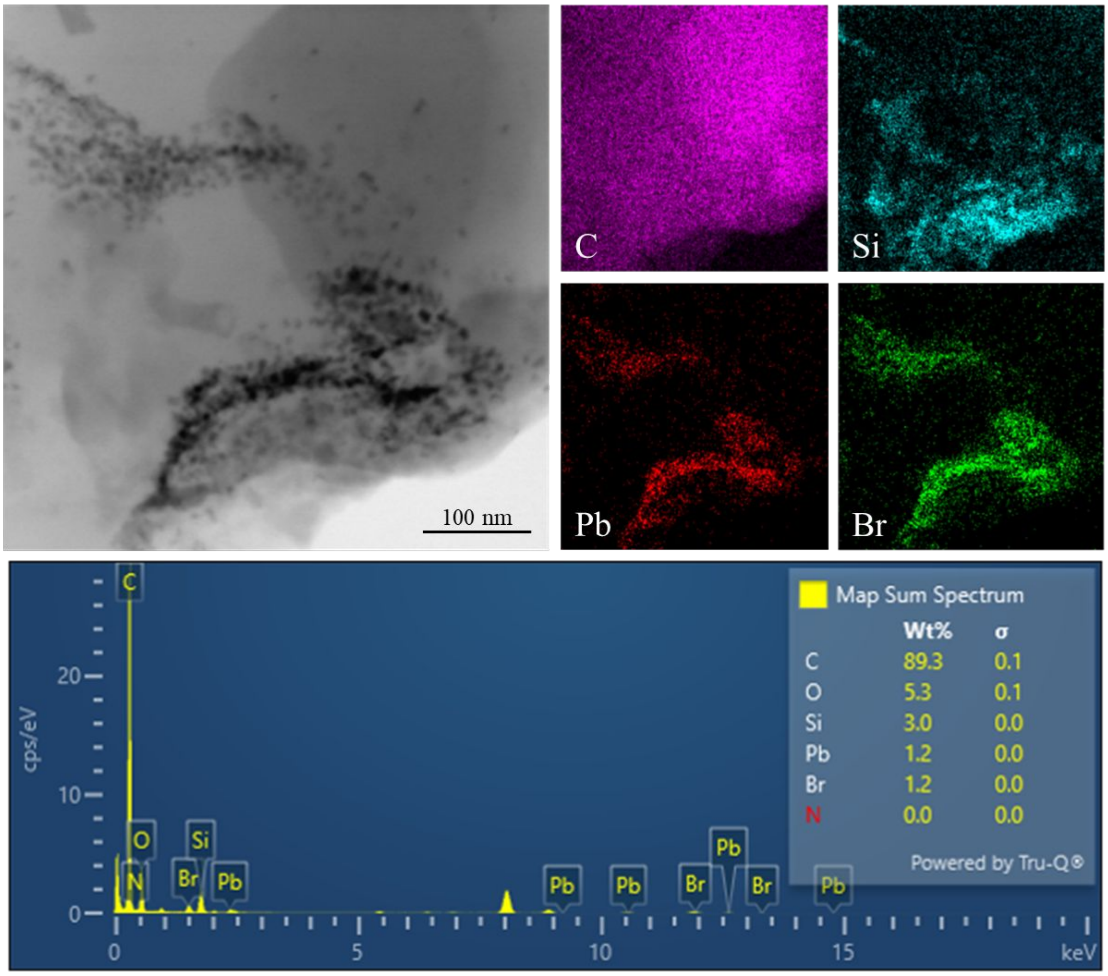

Figure S6. EDS mapping images and elemental composition of $FAPbBr_3@SiO_x@513M$, along with the elemental distribution images for carbon, silicon, lead, and bromine.

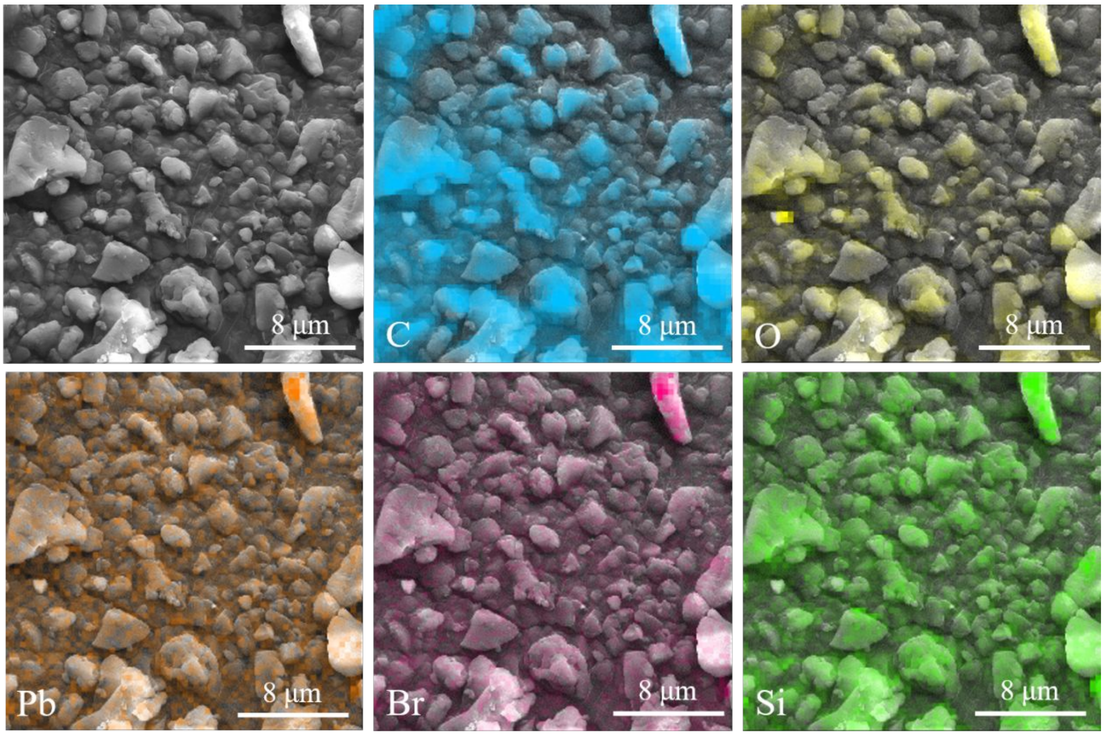

Figure S7. EDS elemental mapping of coarse-ground and un-sieved $FAPbBr_3@SiO_x@513M$ material under a scanning electron microscope (SEM), showing the elemental distribution images for carbon, oxygen, lead, bromine, and silicon.

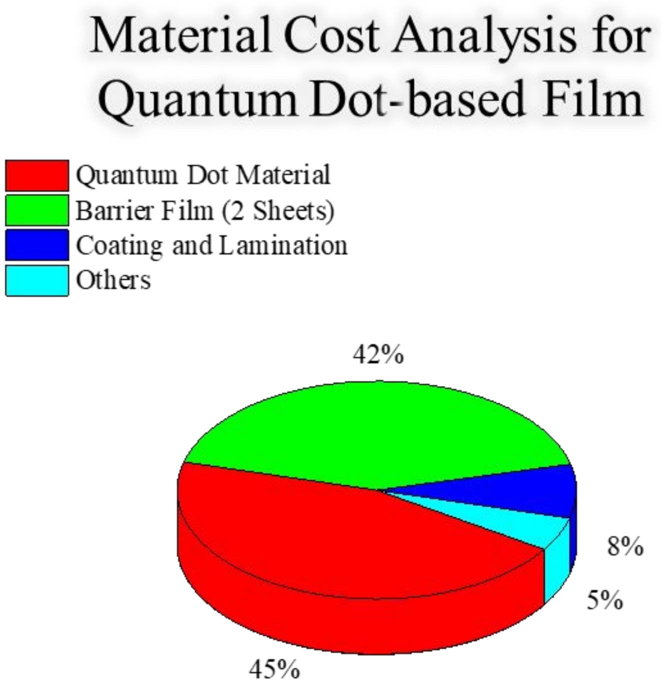

Figure S8. Materials cost analysis for quantum dot-based film pie diagram.

REFERENCES

(1) Wang, S.; Chen, D.; Xu, K.; Hu, J.; Huang, D.; Hong, M.; Zhu, H. Organic Polystyrene and Inorganic Silica Double Shell Protected Lead Halide Perovskite Nanocrystals with High Emission Efficiency and Superior Stability. *Nano Res.* **2023**, *16*, 10507–10514.

(2) Mohapatra, A.; Kar, M. R.; Bhaumik, S. Suppression of Halide Migration and Improved Stability in Double-Coated Cesium Lead Halide Perovskite Nanocrystals for Application in Down-Conversion White-Light-Emitting Diodes. *J. Alloy. Compd.* **2022**, *927*, 166972.

(3) Huang, Y.; Li, F.; Qiu, L.; Lin, F.; Lai, Z.; Wang, S.; Lin, L.; Zhu, Y.; Wang, Y.; Jiang, Y.; Chen, X. Enhancing the Stability of $CH_3NH_3PbBr_3$ Nanoparticles Using Double Hydrophobic Shells of SiO_2 and Poly(vinylidene fluoride). *ACS Appl. Mater. Interfaces* **2019**, *11*, 26384–26391.

(4) Xu, L.; Chen, J.; Song, J.; Li, J.; Xue, J.; Dong, Y.; Cai, B.; Shan, Q.; Han, B.; Zeng, H. Double-Protected All-Inorganic Perovskite Nanocrystals by Crystalline Matrix and Silica for Triple-Modal Anti-Counterfeiting Codes. *ACS Appl. Mater. Interfaces* **2017**, *9*, 26556–26564.

(5) Muchlis, A. M. G.; Lin, C. C. Investigating a Simple and Sustainable Photoluminescence Improvement Approach for a Highly Applicable Perovskite-ZIF-8 Hybrid Material by Using Water. *J. Mater. Chem. A* **2024**, *12*, 26471–26483.

(6) Huo, B.; Yang, J.; Bian, Y.; Wu, D.; Feng, J.; Zhou, J.; Huang, Q.; Dong, F.; Tang, X. Amino-Mediated Anchoring of $FAPbBr_3$ Perovskite Quantum Dots on Silica Spheres for Efficient Visible Light Photocatalytic NO Removal. *Chem. Eng. J.* **2021**, *406*, 126740.

(7) Tabassum, M.; Zia, Q.; Li, J.; Khawar, M. T.; Aslam, S.; Su, L. $FAPbBr_3$ Perovskite Nanocrystals Embedded in Poly(L–lactic acid) Nanofibrous Membranes for Enhanced Air and Water Stability. *Membranes* **2023**, *13*, 279.
